# A Case Report Emphasizing an Early Approach in a Patient With Diffuse Axonal Injury

**DOI:** 10.7759/cureus.52750

**Published:** 2024-01-22

**Authors:** Mansee S Dangare, Akshaya Saklecha, Pallavi Harjpal

**Affiliations:** 1 Department of Neuro-Physiotherapy, Ravi Nair Physiotherapy College, Datta Meghe Institute of Higher Education and Research, Wardha, IND

**Keywords:** neurophysiotherapy, early approach, quality of life, traumatic brain injury, rehabilitation, diffuse axonal injury

## Abstract

Diffuse axonal injury (DAI) is a severe and frequently life-altering form of traumatic brain injury that is brought on by forces of rapid acceleration as well as deceleration impacting the brain. DAI primarily stems from mechanical forces that lead to the widespread disruption of axons throughout the brain. Unlike focal injuries that affect a specific brain region, DAI manifests as multifocal axonal damage, often impairing vital neural connections. This injury occurs due to shear and tensile forces during traumatic events, such as car accidents, falls, and sports-related incidents. This current case report includes a 19-year-old male who had a fall from his bike and was hospitalised with brain trauma. A Magnetic resonance imaging (MRI) scan was done, which revealed a case of DAI, and a computed tomography (CT) scan of the brain revealed the extra-calvarial soft tissue swelling in the left parietal region. Small haemorrhagic contusions involved the right ganglio-capsular region. Several integrative techniques, including joint approximation, proprioceptive neuromuscular facilitation (PNF) rhythmic initiation, D1 flexion-extension, and patient education, were used to manage the patient. The patient's development was evaluated using outcome measures, such as the functional independence measure (FIM) and the Glasgow coma scale (GCS). Thus, we conclude that completing physiotherapy exercises consistently helps patients achieve their highest level of functional independence and also enhances their quality of life.

## Introduction

Diffuse axonal damage (DAI), also known as shear injury or traumatic axonal injury, is intracranial damage produced by the brain's neurons, decelerating or accelerating rapidly and repeatedly [1]. It appears to be connected to comatose following traumatic brain injury (TBI) and is believed to be present in almost all individuals who lose consciousness following a vehicle accident [2]. DAI was observed in 69% of overall mild TBI patients, as well as 89% of serious TBI individuals. DAI with a grade 3 traumatic lesion in the brainstem in areas typically of DAI (dorsolateral quadrant of the upper brainstem, superior cerebellar peduncles, with or without lesions in the lobar white matter) was observed in 30% of the people having serious TBI and 20% of individuals with mild TBI [3]. The brain experiences a variety of forces throughout a TBI, including compressive, tensile, and rotational stresses. The brain's inertia causes the cervical column to become dissociated from its relative motion, so when the head moves quickly throughout trauma without suffering a major physical blow, translational, rotational, and angular accelerations can cause damage to axons or blood vessels to expand, primarily in instances of acceleration due to rotation [4,5]. In addition to primary axotomy caused by mechanical forces, DAI is caused by a slow series of pathological modifications in the axon's molecules and cells that follow the first shear stress at the location of injury [6].

Cognitive deficits, diminished memory, disorientation, and agonizing headaches are diagnostic symptoms and signs of DAI. Despite their catastrophic consequences, multiple studies have connected these damages to major neurological conditions [7]. At present, the primary goal of rehabilitation and physical therapy for patients with brain injuries treated in intensive care units (ICUs) is mainly on the body's functions and organization. Examples of these include oxygen therapy, low-dose endurance and power learning, stimulation treatment, breathing treatment, and passive-assistive activity for contraction prevention. Several of the treatments deal with tasks such as performing self-care or activities of daily living (ADL) learning, along with moving into a wheelchair, sitting posture, or standing [8].

Rehabilitation professionals have used a wide range of strategies to aid in the patient’s rehabilitation who suffered traumatic brain injuries. They focus on specific therapeutic concepts that are endorsed by separate physiotherapy [9]. The incorporation of freshly developed technology, such as Nintendo's Wii and Wii Fit virtual reality (VR) gaming systems, which are sold economically, might also have an impact on intervention material. These engaging and easily available VR video game systems employ motion-sensing technology that recognizes direction and velocity, enabling the kid to manipulate gaming through their movements and postures. These games encourage players to extend or intensify their practice sessions by offering information and repeating realistic challenges on a regular basis [10]. The major goals of therapy are frequently muscular improvement, prevention of subsequent injury such as pneumonia or stiffness, and recovery of consciousness and sensorimotor viewpoint. The primary goal is to get the maximum degree of movement and self-care capability [11].

## Case presentation

Patient information

A 19-year-old male patient had a fall from a motorbike while he was returning to his home. After the accident, the patient was brought to a local private clinic in Chandrapur, with a GCS score of E1V1M4. He was hospitalized for five days after experiencing a loss of awareness without any prior history of bleeding or vomiting. He underwent a magnetic resonance imaging (MRI) scan, which confirmed it to be a case of DAI, and a CT scan revealed that small haemorrhagic contusions were seen involving the right gangliocapsular region of grade 3. He was managed conservatively and referred to a tertiary healthcare center where the patient was referred to the physiotherapy intensive care unit. The patient was intubated, and he was on a mechanical ventilator with mode-continuous mandatory ventilation, positive end-expiratory pressure (5 cm H20), and a fraction of inspired oxygen (70%) and received further treatment.

Clinical findings

On neurological examination, the patient was conscious with a GCS score of E1VTM4 and disoriented. Before beginning the examination, verbal consent was obtained from the patient's relative. He was examined while lying supine. On inspection, it was observed that the patient was seen in a supine lying position with the head end elevated to 30 degrees. The patient's hands and legs were extended and slightly abducted, abrasions over the bilateral knee joint, and Ryle's tube and Foley's catheter in situ. On the day of examination, the GCS score was 5/15 E1VTM4, and the patient was vitally stable. On palpation, the local skin temperature was raised. The muscle tone is shown in Table 1 and reflexes in Table 2. The complete incident's timeframe is depicted in Table 3.

**Table 1:** Muscle tone (tone grading system) of the upper and lower extremities TGS: Tone Grading Scale; 1+: Decreased tone, 2+: Normal tone, 3+: Increased tone

Muscle tone	Right side	Left side
Upper limb	2+	1+
Lower limb	2+	1+

**Table 2:** Reflexes of the upper and lower extremities +: Diminished reflex, ++: Normal reflex, +++: Exaggerated reflex

Reflexes	Right side	Left side
Biceps	++	++
Triceps	++	+
Supinator	++	+
Knee	++	+
Ankle	++	+
Plantar	Flexors	Flexors

**Table 3:** Timeline of events AVBRH: Acharya Vinobha Bhave Rural Hospital

Incidents	Occurrence
Fall from bike	04/10/2023
Visited a private hospital in Chandrapur	04/10/2023
Discharge from a hospital in Chandrapur	09/10/2023
Date of admission in AVBRH	09/10/2023
The date of physiotherapy started	15/10/2023

Diagnostic assessment

**Figure 1:**
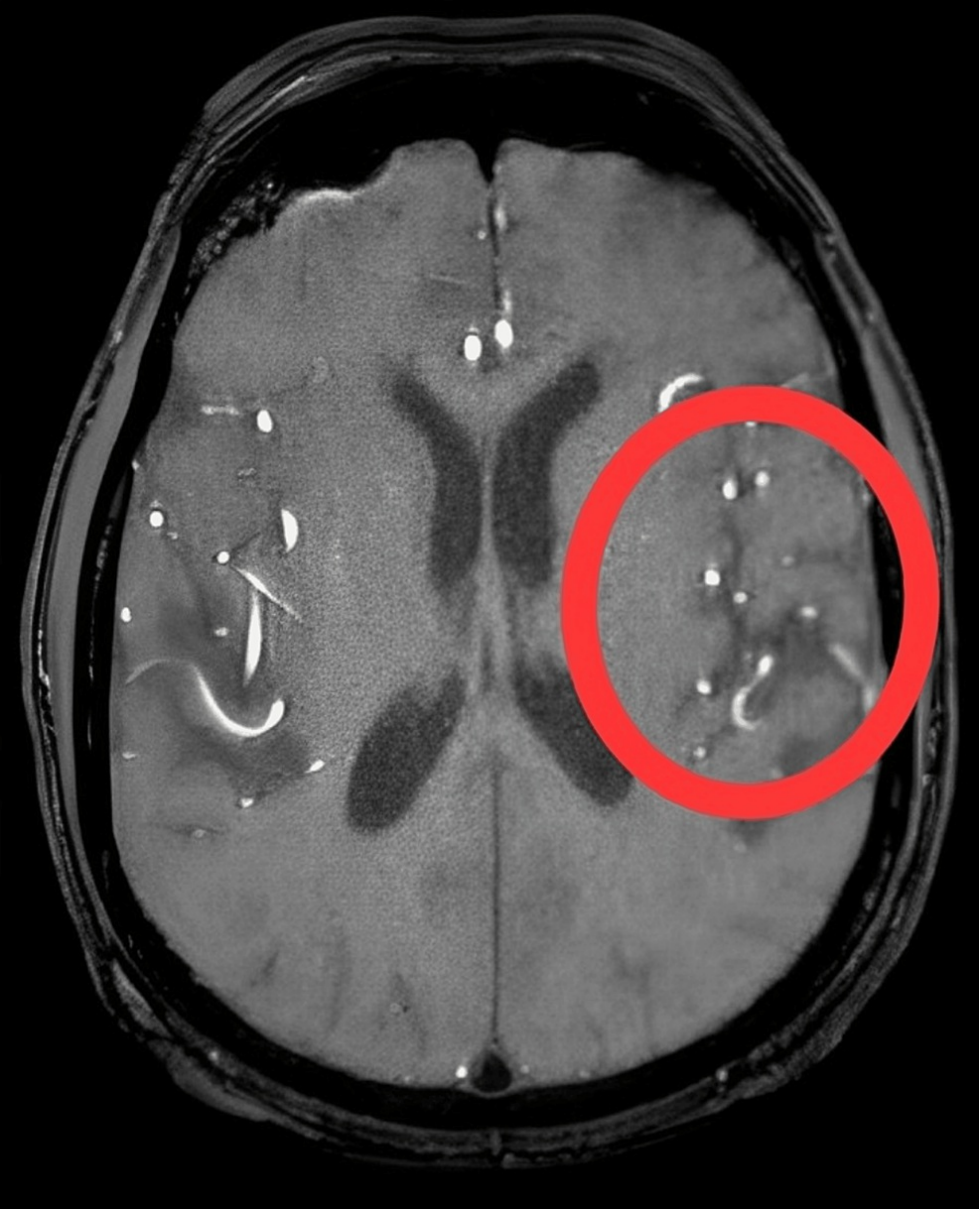
MRI of the brain The circled part indicates multiple foci blooming on SWI noted diffusely scattered through brain parenchyma involving subcortical, cortical regions, deep white matter, bilateral gangliocapsular regions, brainstem, and cerebellum suggestive of diffuse axonal injury. MRI: Magnetic resonance imaging, SWI: Susceptibility-weighted imaging

MRI reveals multiple foci of blooming on susceptibility-weighted imaging (SWI) noted diffusely scattered through brain parenchyma involving subcortical, cortical regions, deep white matter, bilateral gangliocapsular regions, brainstem, and cerebellum suggestive of DAI, as shown in Figure 1.

Medical management

Medicines such as Tab. cognilong plus, Tab. lasix, Tab. flucanazole, Tab. nicardia, Tab. levepsy, Tab. doxycycline and dosage, along with their duration, are given in Table 4.

**Table 4:** Dosage and duration of medication Tab: tablet; mg: milligrams; BD: twice a day; OD: once a day; TDS: thrice a day

Serial. No.	Medication	Dosage	Duration
1.	Ascorbic Acid+ Cerebroprotein Hydrolysate +Cholecalciferol +Folic Acid +Methylcobalamin +Piracetam +Pyridoxine +Thiamine +Tocopherol	20 mg	BD
2.	Tab. Lasix	15 mg	OD
3.	Tab. Nicardia	20 mg	TDS
4.	Tab. Levepsy	500 mg	BD
5.	Tab. Doxycycline	10 mg	BD

Therapeutic intervention

**Figure 2:**
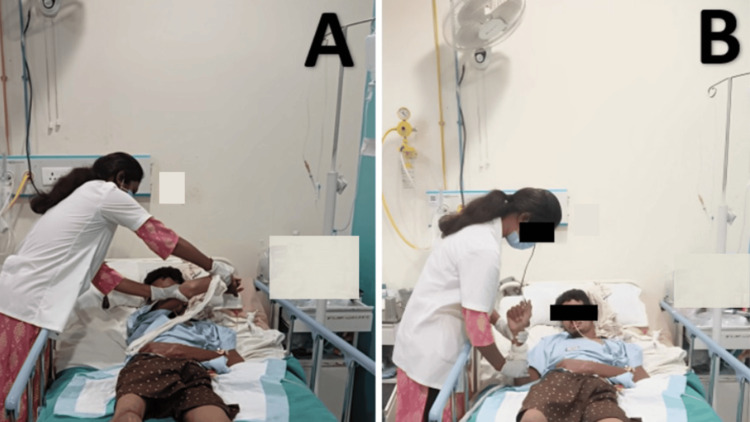
Physiotherapist performing PNF PNF: Proprioceptive neuromuscular facilitation (A) PNF rhythmic initiation D1 flexion-extension to upper extremity, (B) joint approximation to upper extremity

The goal-oriented physiotherapy protocol is given in Table 5. Physiotherapy sessions included proprioceptive neuromuscular facilitation (PNF) rhythmic initiation D1 flexion-extension to the left upper and lower extremity (Figure 2A) and the joint approximation to the upper limb and lower limb (Figure 2B).

**Table 5:** Goal-oriented physiotherapy protocol ROM: Range of motion; PNF: proprioceptive neuromuscular facilitation

Goals	Therapeutic intervention	Treatment protocol
Patient education	A patient relative was informed about their disease as well as the importance and advantages of physical rehabilitation. Obtaining the family members' confidence and permission.	Education was given to caretakers regarding the significance of appropriate body alignment.
To normalise muscle tone	PNF rhythmic initiation D1 flexion-extension to left upper and lower extremity.	A single set of ten repetitions two times each day.
	Joint approximation to upper extremity and lower extremity.	
To improve left upper limb and lower limb range of motion exercises	Passive ROM exercises to left upper and lower extremity.	A single set of ten repetitions, two times each day.
To improve right upper limb and lower limb range of motion exercises	Active assisted ROM exercises to the right upper extremity and lower extremity.	A single set of ten repetitions, two times each day.
To ensure good ventilation	Deep breathing exercises and pursed lip breathing exercises.	A single set of ten repetitions, two times each day.
Improve airway permeability	Manual chest techniques (percussion and vibrations), suctioning and positioning.	Changing positions every two to three hours and manual methods for 10 repetitions.
Bed mobility	Supine - side lying - bedside sitting with maximal assistance.	2 times a day for 1 week.
To avoid pressure sores resulting from extended immobilisation	Manual positioning, air bed provided.	Positioning for every 2-3 hours.
Getting out of bed with a wheelchair	To encourage the patient's mobility and raise their level of attentiveness.	Every day for 1 hour.

Follow-up and outcome measures

The pre-intervention outcome taken on day one and follow-up taken on the sixth week of intervention are given in Table 6.

**Table 6:** Pre- and post-intervention physiotherapy outcome measures RLA: Ranchos los amigos scale; ICU: Intensive care unit; TBI: Traumatic brain injury Glasgow coma scale: 11 - Moderate TBI, 15 - Mild TBI; TGS: Tone Grading Scale - 1+: Decreased tone, 2+: Normal tone, 3+: Increased tone; Functional independence measure: 1 - Total assistance, 6 - Modified independence; RLA scale: 2 - Generalized reflex response to pain, 6 - Confused/appropriate. Inconsistently orientation to time, place, person, consistently follows simple direction; ICU mobility scale: 0 - Nothing (lying on bed), 6 - Marching on spot (at bedside)

Serial. No.	Outcomes measured	Pre-intervention	Post-intervention
1.	Glasgow Coma Scale	11/15	13/15
2.	Tone grading scale	1+	2+
3.	Functional Independence Measure	1/7	6/7
4.	RLA scale	2/10	6/10
5.	ICU mobility scale	0/10	6/10

## Discussion

In this case, the patient suffered from serious diffuse axonal damage, which was treated conservatively using appropriate drugs, physical therapy, and other supportive therapies. The treatment procedure's goals were defined by first thoroughly teaching the patient and his family about his illness and the necessity of physiotherapy for his recovery.

Sometimes, the physiotherapist may combine various additional active or passive tissue "stretching" methods (such as appropriate posture, passive movements, and weight bearing) with a range of splints, customised orthoses, and plaster casts. Treatment goals usually involve lengthening shortened soft tissues, as well as inhibiting/reducing increasing muscular tone. Thus, in order to achieve this goal, early rehabilitation included chest-clearing activities, mild range-of-motion exercises, and facilitative methods for returning muscle tone to normal. Further, stretching methods were supplied to increase joint flexibility and avoid muscular tightness, and strengthening activities were provided progressively to enhance the endurance and strength of the muscles. Histopathological abnormalities in the lobar white matter, corpus callosum, and dorsolateral parts of the brainstem have been associated with higher trauma damage [12].

Sensorimotor reactions, including neuroplasticity and functional improvements, found throughout therapy after diffuse axonal injuries, are important considerations for physicians to consider. These mechanisms are connected to the level of cardiovascular exercise [13,14]. Neuroplasticity can be useful when it comes to improved functioning or maladaptive when it comes to diminished or abnormal performance. Rehabilitation's job is to steer the nervous system towards responsive neuroplasticity by evaluating and organizing sensorimotor information appropriately [15]. Research results promote the importance of changed axonal calcium homeostasis within the process of axonal secondary destruction, and they indicate that calcium channel blockers, which may reduce secondary harm, in addition to additional mechanisms suggested in secondary damage, may be focused on as treatments [16,17]. Outside the cell, Ca2+ is the primary cause of reactive oxygen species (ROS)-mediated degeneration of axons. Extracellular Ca2+ elimination, instead of blocking mitochondrial Ca2+ discharge, is an effective method for reducing intracellular calcium and limiting spheroid development [18]. Oxidative stress, a condition that damages the natural antioxidant defence systems, plays a crucial role in the subsequent processes that contribute to neuronal degeneration. Increased defence mechanisms by external antioxidants may be neurologically protective, especially if provided during the neuroprotective period frame [19,20].

## Conclusions

DAI is a severe and frequently fatal kind of brain injury caused by trauma that causes extensive destruction of axons throughout the brain. DAI can result from various types of accidents and head trauma, and its effects can vary. TBI induces persistent malfunction and apoptosis of neuronal cells both close and far from the area of damage. Physiotherapy is an integral component of the multidisciplinary approach to managing DAI. It plays a significant role in helping the patient regain his physical and cognitive abilities and enhancing the quality of life in DAI.
